# Torsional behavior of Ni-rich NiTi alloys obtained by powder metallurgy and hot deformation

**DOI:** 10.1038/s41598-024-79545-2

**Published:** 2024-11-18

**Authors:** Sergey Volodko, Galina Markova, Sergey Yudin, Darya Permyakova, Ivan Alimov, Evgeny Evstratov, Dmitry Moskovskikh, Alexander Khort, Anatoly Kasimtsev

**Affiliations:** 1 Moscow Polytechnic University, Moscow, Russia; 2 Tula state university, Tula, Russia; 3 LLC Metsintez, Tula, Russia; 4 A. Baikov Institute of Metallurgy and Materials Science, Moscow, Russia; 5 National University of Science and Technology MISIS, Moscow, Russia; 6 KTH Royal Institute of Technology, Stockholm, Sweden

**Keywords:** Structural materials, Metals and alloys

## Abstract

The effects of severe plastic deformation on NiTi alloys’ structure and properties have been extensively studied over the past decades. However, there is a notable lack of systematic data regarding the impact of industrial hot deformation techniques on these alloys. This gap arises from challenges in manufacturing processes related to the unevenness of ingots produced by casting technologies. This study investigates the effects of hot rotary swaging, extrusion, and radial shear rolling on the martensitic transformation, shape memory effect, superelasticity, and damping capacity of NiTi Ni-rich alloys fabricated through powder metallurgy. The properties were investigated under torsional load on wires prepared by spark eroding from deformed rods. Our findings indicate that samples after rolling and extrusion exhibit a superelastic strain of 14 ± 0.5% attributed to a high yield stress of approximately 600–800 MPa and torsional testing providing the material to be fully involved in recovery process. Samples after rolling and swaging demonstrate a high level of reversible strain with a one-way shape memory effect ranging from 5 to 7%. Conversely, extrusion, due to the inhomogeneity of resulting workpieces, induces a complex, multi-stage martensitic transformation that undermines the shape memory effect. Furthermore, all deformation methods except extrusion contribute to increased alloy homogeneity, resulting in a narrower temperature range for martensitic transformations. Rotary swaging notably increases the height of an internal friction peak from 0.015 to 0.045 compared to the undeformed material, whereas rolling gives the lowest value of 0.012 among others. This study provides valuable insights into how hot thermomechanical processing influences the properties of NiTi alloys and shows that powder metallurgy combined with hot deformation can be considered an alternative approach for achieving high functional properties of these alloys.

**Supplementary Information:**

The online version contains supplementary material available at 10.1038/s41598-024-79545-2.

## Introduction

NiTi is an intermetallic compound that can endure severe plastic deformations and accumulate large strains without failure. Many studies are devoted to the effect of plastic deformation production methods on the structure and properties of NiTi-based alloys and are usually carried out using severe plastic deformation methods such as angular extrusion (ECAP), high-pressure torsion (HPT), etc^1–3^. These methods ensure the appearance of an amorphous structure, thereby it is possible to obtain nano- or ultra-fine grains by low and intermediate aging and have excellent mechanical and functional properties. However, shape memory effect (SME), including superelasticity (SE), mostly depends on the yield stress of a matrix, where dislocation gliding should not occur in order to approach theoretical crystallographic deformation.

Sometimes such extreme deformation conditions are not necessary to achieve good SME and SE levels. There is very little data on the effect of industrial hot deformation techniques on the characteristics of NiTi alloys^4–8^, arguably due to the difficulties in the manufacturing process connected to the inhomogeneity of massive ingots obtained by casting technologies. It is shown that hot deformation leads to grain refinement via dynamic recrystallization; however, no information on the functional properties of NiTi alloys are presented. Consequently, the aforementioned studies did not provide insights into understanding the effect of hot deformation on functional characteristics of NiTi alloys and are focused on texture and structure evaluation with no connection to SME and SE.

NiTi alloys can be used for civil construction to increase the stability and improve damping capacity of concrete structures and buildings^9,10^. There, massive rods having high functional characteristics are necessary and deformation production methods, such as ECAP and other severe plastic deformation approaches are not suitable due to their limiting productivity and working sizes.

Massive ingots are hard to deform due to dendritic structure. However, powder metallurgy (PM) allows to avoid solidification, and consequently, Ti_2_Ni inclusions should not appear due to dendritic liquation, which requires several remelting to be capable of enduring subsequent plastic deformation. This fact highlights the relevance of PM to obtain high-quality alloys provided optimal technological parameters.

Therefore, the purpose of the study is to assess the effect of hot deformation by industrial processing methods on martensitic transformation and functional properties of binary Ni-rich NiTi alloys and additionally demonstrate the applicability of PM in obtaining shape memory alloys with enhanced functional characteristics.

## Experimental

NiTi alloys were prepared from a TiO_2_ oxide (> 99 wt%) and pure Ni powder (> 99.5 wt%) by mixing them with CaH_2_ (a reducing agent) followed by heating up to 1200 °C and holding for 6 h under an argon atmosphere. The synthesis is described with the following redox reaction:

1 $$Ti{O_2} + {\text{ }}Ni{\text{ }} + {\text{ }}2Ca{H_2} \to {\text{ }}NiTi{\text{ }} + {\text{ }}2CaO{\text{ }} + {\text{ }}2{H_2} \uparrow$$

A CaO compound was removed from the synthesis products with the aid of HCl acid and water by transforming CaO into CaCl_2_, which has a high solubility in water and is easily removed by washing. Rods for deformation were prepared by pressing the powders in an isostatic press under 200 MPa followed by vacuum sintering at 1280–1290 °C under a vacuum of 5∙10^− 3^ Pa. Residual porosity was about 2% for all as-sintered rods. The chemical composition is shown in the supplementary file (Table S1).

The deformation of the as-sintered blanks was carried out by rotary swaging, radial shear rolling, and extrusion. The initial diameters of the rods for swaging, extrusion, and rolling were 15, 33, and 78 mm, respectively. Deformation temperatures were 900 °C for rolling and extrusion, and 900 and 600 °C for swaging. The samples were deformed in air and then cooled to room temperature in air. More detailed information on deformation is provided in the supplementary file. Hereinafter, samples subjected to extrusion, rolling, and rotary swaging will be marked as EX900, R900, RS900, and RS600.

Internal friction (IF) was studied on a reverse torsion pendulum with an electromagnetic system for deforming the sample by torsion and an optical semi-automatic system for recording deformation. Internal friction values were calculated as^11^:

2 $$\:{Q^{ - 1}} = \:\frac{1}{{\pi \:n}}ln\left( {\frac{{{A_i}}}{{{A_n}}}} \right),$$

where A_i_ and A_n_ are the amplitudes of the first and n_th_ oscillations, *n* is the number of oscillations in the selected amplitude range.

The measurements of internal friction were carried out in the free-damping oscillation mode with a heating/cooling rate of ∼ 1 °C at a frequency of ∼1.5 Hz and a deformation amplitude of 5·10^–5^.

As a measure of elastic properties in the low-frequency range, we used the squared value of the frequency of free-damped oscillations (f^2^), proportional to the shear modulus *G*^11^. The squared resonant frequency was calculated using the formula:

3 $$\:{f}^{2}=\:\frac{1}{{\overline{T}}^{2}}\:,$$

where $\bar T$ is the average value of the oscillation period in the selected amplitude interval, calculated based on five measurements. The measurements were carried out on wire samples with a length of 45–55 mm and diameter d = 1 mm. The wires were cut out by an electrical discharge machine from sintered and deformed rods then ground to remove an oxide layer from the surface. The wires were taken along the main axis of a deformed rod. When the wires were attached in the apparatus for internal friction measurements, they were annealed at 250 °C in a fore vacuum (~ 1.3 Pa) for one hour to relieve elastic stresses in the sample to eliminate its effect on martensitic transformation.

The shape recovery characteristics of the studied alloys during torsional load were determined using a setup, which is based on the design of a reverse torsion pendulum and schematically represented in the supplementary file in Figure S1a. The studied samples were identical to those used to measure internal friction. The deformation of the sample by torsion was calculated using the formula^12^:

4 $$\gamma = \frac{{\pi r\varphi }}{{180l}} \cdot 100,$$

where *r* is the radius of a sample, mm; *φ* – twist angle, rad; *l* – a working length of a sample, mm, the error of deformation evaluation is about 0.5%.

To assess the spread of Ni content over the matrix, the energy-dispersive spectroscopy (EDS) was performed on a TESCAN VEGA LMH electron scanning microscope with an attachment for energy-dispersive microanalysis of the Oxford Instruments Advanced AZtecEnergy.

X-ray analysis was conducted on a DRON-3 M diffractometer with a graphite monochromator and Cu K-alpha and Co K-alpha radiations. Dislocation densities were assessed via X-ray method according to the broadening of Bragg’s reflection of B2-phase based on Williamson and Smallman approach^13^ through estimation crystallite size and microstrain. Instrumental broadening was assessed using a coarse grain Ni standard. The dislocation density due to crystallite size and strain in the material was calculated from the crystallite size, root mean square (RMS) microstrain, lattice parameter and the corresponding Burger’s vector of the active dislocation from the equation:

5 $$\:{\uprho\:}\:=\:{({{\uprho\:}}_{\text{D}}\bullet\:{{\uprho\:}}_{\text{S}})}^{1/2}\:,$$

where ρ_D_ = 3/D^2^ (due to crystallite size) and ρ_S_ = k‹ε^2^›/b^2^ (due to strain), where k is the material constant^13^, D is the crystallite size, ε is the root mean square microstain, b is the Burger’s vector. Here a ·$\:<100>$ is used as Burger’s vector used in calculations^14^.

## Results and discussion

The thermoelastic martensitic transformation (MT) contributes to the unique properties of NiTi alloys, and the staging of this phenomenon plays an important role in deformation recovery. To study the forward and reverse martensitic transformation, the method of mechanical spectroscopy, also known as IF, was used. It was shown in^15^ that data on temperature ranges and stages of martensitic transformations obtained by the most common method, differential scanning calorimetry (DSC), and the IF are in good agreement with each other. In addition, it is possible to evaluate the dissipative properties (damping capacity) of a material by IF in low frequency region^16^.

**Fig. 1:**
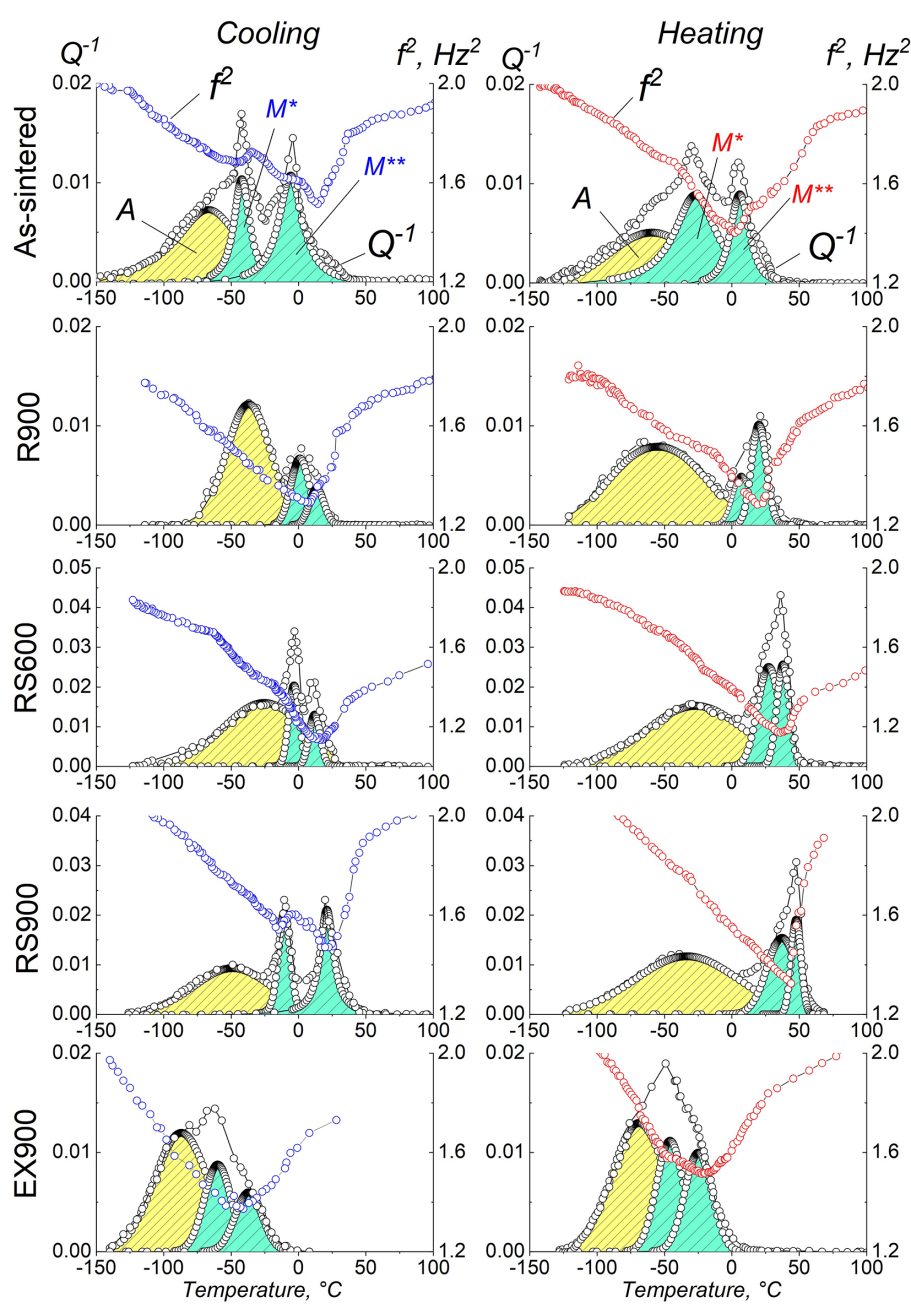
Temperature dependences of IF and f^2^ of the NiTi alloys after all types of deformation (IF background is removed). The spectra on the left and the right were taken at cooling (blue) and heating (red), respectively.

Figure 1 shows the fragments of IF spectra and the square resonant frequency (f^2^) for the as-sintered state and after various types of deformation. The IF spectra are shown after subtracting the background.

The appearance of the obtained spectra of the square resonant frequency and, consequently, the shear modulus (f^2^ ~ G) allows us to obtain information on the stages of martensitic transformations. An f^2^ minimum indicates the occurrence of MT and should coincide with an IF maximum. It can be assumed that if the martensitic transformation occurs according to the B2↔R↔B19’ way, then two minima are observed in the f^2^(t) curve, and two corresponding maxima or one asymmetric maximum are observed in the IF spectrum.

According to Fig. 1, the IF spectra can contain from 1 (EX900) to 3 (RS900) quite broad IF maxima, the origin of which is different. The maximum “A” is of a relaxation nature. In^17,18^, it is assumed that the maximum is due to the relocation of point defects to dislocations and their subsequent pinning. In^19^, it was suggested that the peak is due to several relaxation processes associated with the interaction of dislocations and pinned vacancies. The authors agree that the nature of that maximum is relaxation and is associated with the presence of twinning dislocations in the martensite structure. The other two maxima (M* and M**) are due to the thermoelastic MT.

**Fig. 2:**
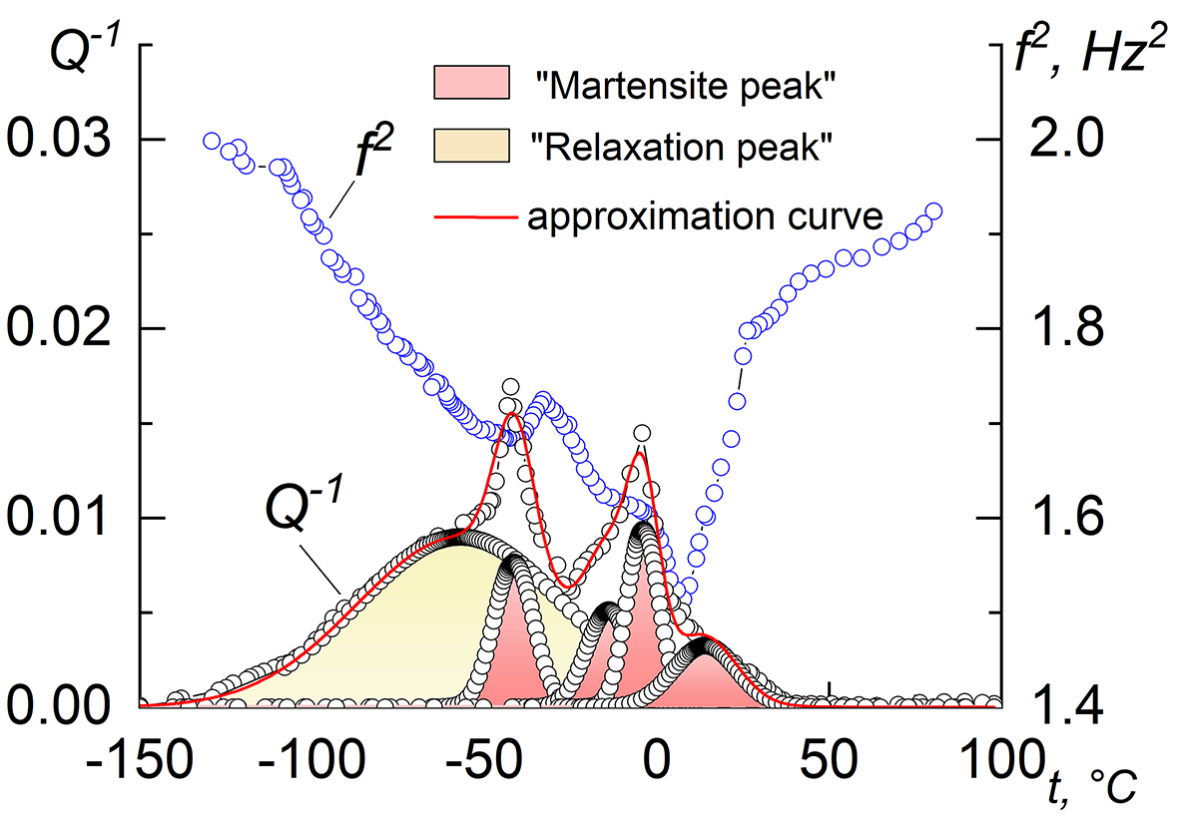
Temperature-induced IF during cooling for the as-sintered NiTi alloy.

However, in NiTi alloys enriched in nickel, two-, three- and four-stage transformations can be observed, consisting of consecutive transformations B2↔B19’, B2↔R, and/or B2↔R↔B19’^20–23^. For example, in the f^2^(t) dependence of the as-sintered sample, even 5 peaks can be distinguished in the IF spectrum, 4 of which most likely belong to martensitic transformations, and the wide peak A is relaxation (Fig. 2). In this case, the approximation curve best describes the experimental dependence of IF. Consequently, in NiTi powder alloys, a multi-stage MT can be observed, and individual peaks on the IF curve may not be associated with the classical B2↔R↔B19’ transformation, but may be independent transformations B2↔B19’, B2↔R or B2↔R↔B19’. A discussion on it is presented below when discussing the shape memory effect.

The nature of phase precipitation during the aging of Ni-rich NiTi alloys is significantly influenced by the grain size of the B2 phase, the degree of supersaturation of the matrix with nickel, and the time and temperature of aging. In general, in cast materials, a cascade of several sequential transformations occurs due to the inhomogeneous precipitation of the metastable Ti_3_Ni_4_.

In the case of powder alloys, “multi staging” may be associated with a non-uniform distribution of elements. EDS analysis has shown that the variation in the nickel content in the matrix of the as-sintered alloy can reach 1.6 at% (Figure S3 in the supplementary file), depending on the scanning location, which, in addition despite a simple shift in MT temperatures due to changes in nickel content, can cause varying the degree of supersaturation of the matrix with nickel and lead to local inhomogeneous precipitation of metastable Ti_3_Ni_4_. Such precipitates have been observed in the sample after radial shear rolling by transmission electron microscopy (Figure S2 in the supplementary file). Previously, the same was observed in Ti-50.7Ni %at alloy^6^.

The analysis of the experimental data depicted in Fig. 1 shows that upon cooling, the process of martensitic transformation is often accompanied by the formation of two minima in the f^2^(t) curve. However, asymmetric IF maxima are observed in almost all spectra.

At heating, in most cases, one minimum is present in the f^2^(t) curve and an asymmetric “martensitic” maximum of IF, which can be observed in all IF spectra of the samples after deformation. In turn, the asymmetric IF peak both during cooling and heating of the samples after deformation is probably a superposition of two peaks from several martensitic transformations and can be divided into partial peaks using the approximating analytical functions of Gauss, Lorentz, Voight, Pearson or their variations as it is done in Fig. 1.

The smallest width of an IF peak (temperature interval) and, most likely, the associated MT interval, is observed in samples RS600, RS900, and R900. Accordingly, rotary swaging and radial shear rolling lead to a narrowing of the IF peaks and temperature ranges of MT, thus reducing the possible temperature range of shape recovery, which is important from a practical point of view. This trend is clearly visible in the IF at heating. To use shape memory alloys as, for example, actuators, it is necessary to have a narrow range of shape recovery, and, accordingly, a narrow range of reverse MT, at a level of ~ 30–50 °C. Table 1 shows the MT temperatures determined from the IF spectra.

**Table 1:** MT temperatures of the NiTi alloys calculated from IF.

		M_s_ ,°С	M_f_ ,°С	A_s_, °С	A_f_, °С	A_f_-A_s_, °С	M_s_-M_f_, °С	|A_f_ - M_s_|, °С
Powder 1	As-sintered	33	-56	-62	40	102	89	7
	R900	25	-16	-9	37	46	41	12
	RS600	28	-17	9	50	41	45	22
	RS900	42	-27	10	57	47	69	15
Powder 2	As-sintered	-2	-97	-41	26	67	95	28
	EX900	-12	-84	-68	2	70	72	14

Notation: *M*_*s*_ and *M*_*f*_ are the temperatures of the onset and end of forward MT at cooling; *A*_*s*_ and *A*_*f*_ are the temperatures of the onset and end of reverse MT at heating.

**Fig. 3:**
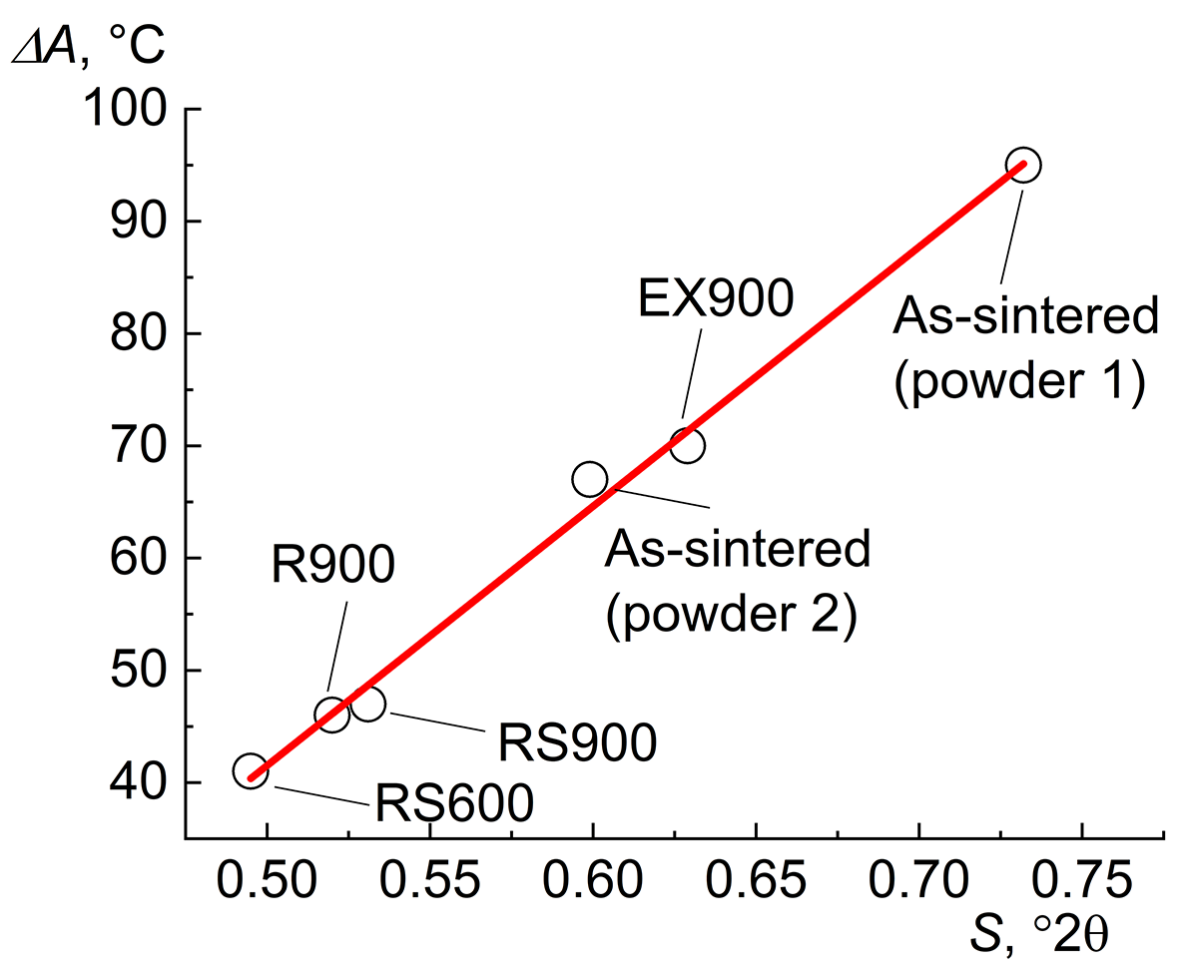
The effect of the integral broadening (S) of the (101) peak of the high-temperature B2 phase on the temperature interval of reverse martensitic transformation (∆A).

It is worth noting that the deformation can lead to a change in MT temperatures; however, no patterns are found except for the influence of deformation on the width of MT temperature intervals. The width of the reverse temperature interval *(∆A = A*_*f*_*-A*_*s*_*)* correlates with the integral broadening of the Bragg reflection peak from the (101) plane of the B2 phase (S) (Fig. 3). The larger S, the wider the interval of reverse martensitic transformation (∆A) and vice versa.

Many factors contribute to the integral broadening of X-ray peaks, such as the size of crystallites, dislocation density, chemical heterogeneity, etc. Instrumental broadening was not taken into account here, since all experiments were performed on the same equipment under the same conditions, so it is the same in all cases. The grain size in all materials studied is more than 1 μm, so their contribution to broadening is minimal. Dislocation density in sample RS600 (2.5*10^10^ cm^− 2^) and RS900 (1*10^10^ cm^− 2^) assessed by X-ray method by using a coarse grain Ni standard are virtually the same. Accordingly, this contribution to the broadening can be neglected. Therefore, to first approximation, the greatest contribution to the broadening is due to chemical heterogeneity, which is likely formed at the stage of calciothermic synthesis and is inherited in the sintered workpiece. A decrease in the broadening after rolling and swaging compared to the as-sintered sample (powder 1) indicates the redistribution of the main elements in the workpieces (homogenization). This is accompanied with a decrease in the temperature range of reverse martensitic transformation for samples R900, RS600, and RS900 (Fig. 3). Extrusion has little effect on the integral broadening compared to the as-sintered state (powder 2), and the interval of the reverse martensitic transformation (∆A) practically does not change.

To confirm that rolling and swaging promote homogenization, energy-dispersive analysis (EDS) of a metallic matrix was performed. The EDS results have confirmed that rolling and swaging lead to homogenization. The spread of Ni concentration in the as-sintered alloy is 1.6 at% (Figure S3) and that for samples RS900 and R900 is 0.8 and 0.6 at% (Figures S5 and S6), respectively. As the chemical heterogeneity and integral broadening (S) decrease, the width of the MT interval (∆A) decreases. Consequently, rotary swaging and radial shear rolling lead to the homogenization of the NiTi alloy, as demonstrated by the ∆A(S) dependence.

Most likely this is due to the fact that extrusion is carried out in one pass in several seconds, resulting in no redistribution of elements, whereas, for instance, rolling was conducted in 9 passes and heated between them to deformation temperature. This led to the equalization of chemical composition within a metal matrix. Thus, it is expected that in samples with the narrowest IF peaks (R900, RS600, and RS900) and the smallest integral broadening (S), the smallest temperature range of shape recovery will be observed.

**Fig. 4:**
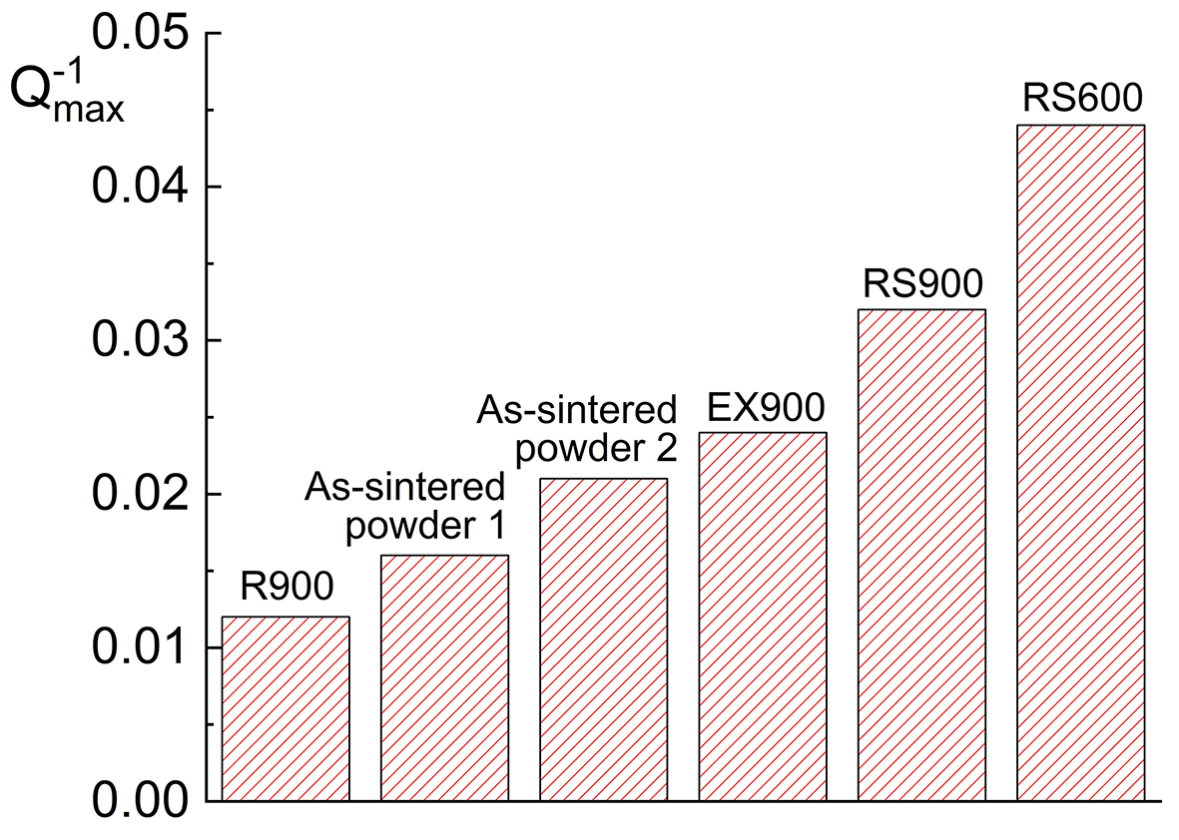
Maximum value of IF of the NiTi alloys before and after deformation.

Another important functional property is the damping capacity, which is directly proportional to the value of IF. The highest IF values (the height of the IF peak) are observed in samples after swaging at 600 and 900 °C (Fig. 4). It may be related to decreasing twin size after deformation and requires additional experiments and accurate scanning electron microscopy investigations. The decrease in twin size and the increase in twin density with increasing deformation strain by hot rolling were observed previously for a cast NiTi alloy^24^. In case rolling in the current study, the lowest IF peak compared to the others may be associated with texture along which martensitic shear stress (stress for martensite reorientation) is higher than for other samples with its own texture, which requires additional experiments. NiTi alloys usually exhibit IF values for thermoelastic martensitic transformation around 0.025-0.1 under similar testing parameters as used in the current work^25^. Therefore, IF values obtained are comparable with cast NiTi alloys.

The main characteristics of shape memory alloys, the shape memory effect and superelasticity, were determined in the martensite region (according to IF spectra) and in the region where only the B2 phase exists. A study of the effect of preliminary (pre-set) deformation on the characteristics of shape recovery shows that at T_pre−set_ = A_f_ +15 °C (B2-phase state), induced deformation returns immediately after the load is removed which says about the alloy exhibiting superelasticity. For the as-sintered samples, RS600, and RS900, the critical strain, after which irreversible (non-recovered) deformation appears, is 12%, and for EX900 and R900–14%. Further heating does not lead to additional recovery. The yield strength of samples EX900 and R900 is 25–40% higher than in other cases (Table S2 in the supplementary file), which provides the greatest resistance to the development of deformation by sliding, which explains their increased functional properties (superelasticity).

**Fig. 5:**
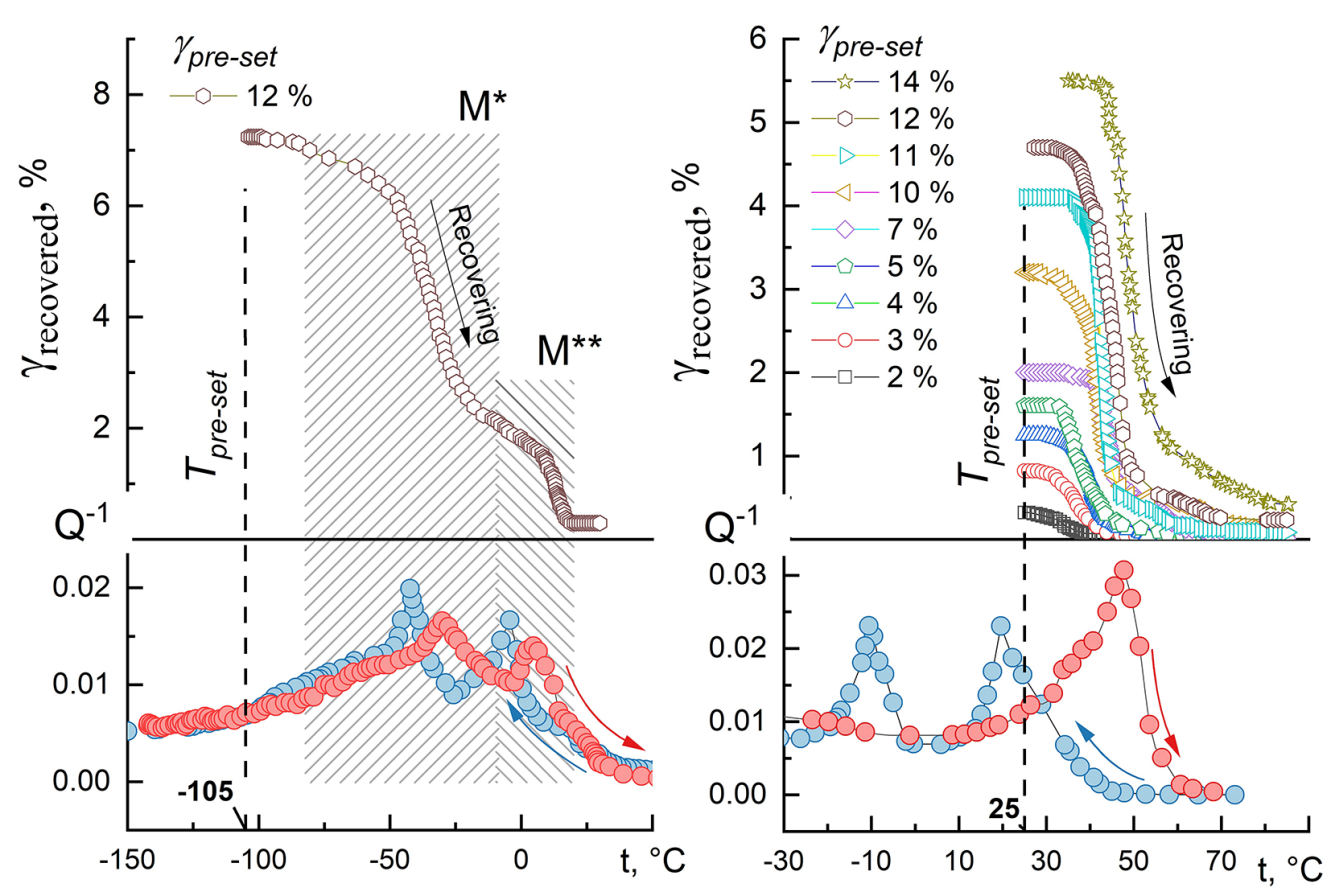
Shape recovery curves at heating of the as-sintered sample at a pre-set strain of 12% and the corresponding fragment of IF (**a**); Shape recovery curves at heating for RS900 at a pre-set strain from 2 to 14% and the corresponding fragment of IF (**b**).

To study the effect of pre-set strain on the one-way shape memory effect, the deformation of the samples was carried out at a temperature close to the temperature of the beginning of the reverse MT (Fig. 5).

Typical shape recovery curves upon heating are shown in Fig. 5. One can observe that the deformation recovered at heating is much lower than the pre-set one. This is due to the superelasticity effect because some number of B2-phase remains in the structure. For example, for sample RS900 with a pre-set strain of 14%, about 8.5% is recovered by superelasticity immediately after the unloading (not shown in Fig. 5), which indicates the presence of a high-temperature B2 phase in the material. The temperature range of shape recovery upon heating is in good agreement with the position of the IF peaks corresponding to the reverse MT. In the case of “broad IF peaks” (EX900, the as-sintered samples), shape recovery occurs in a wide temperature range in two stages, the beginning and end of which coincide with the position of the MT peaks in the IF spectra (M* and M**) during heating. The strain recovered at stage 1 is ~ 5%, and at stage 2 ~ 2%. This indicates that these transformations can be either two B2↔B19’ transformations or two B2↔B19’ and B2↔R↔B19’, or a combination of them, but not B19’↔R and R↔B2 transformations, as it is observed in cast alloys, since the amount of reversible lattice deformation during the B2↔R transformation does not exceed ~ 1%, and for B2↔B19’ – ~ 11% ^26^. Accordingly, the recovery of accumulated strain at both the first and second stages most likely occurs due to several individual B2↔B19’ and/or B2↔R↔B19’ transformations or their combinations. This is also evidenced by several IF maxima and f^2^(t) minima in Fig. 2, which, according to general concepts, cannot be a consequence of a single B2↔R↔B19’ transformation. However, in samples with “narrow peaks” of IF, for example, RS900, RS600, R900, shape recovery occurs without a clearly expressed two-stage process (Fig. 5), and only one maximum is observed in the IF during heating, the position of which agrees well with the temperature range of shape recovery.

A practically important characteristic of the shape memory effect is the critical deformation, starting from which the unrecovered part of the deformation appears due to irreversible sliding. In this sense, this value is represented by the shape recovery coefficient K:

6 $$\:K=\frac{{\gamma\:}_{pre-set}-{\gamma\:}_{irr}}{{\gamma\:}_{pre-set}}*100,$$

where $\:{\gamma\:}_{pre-set}$ is the induced strain by torsion; $\:{\gamma\:}_{irr}$ is the unrecovered strain after the manifestation of superelasticity and the one-way shape memory effect.

**Fig. 6:**
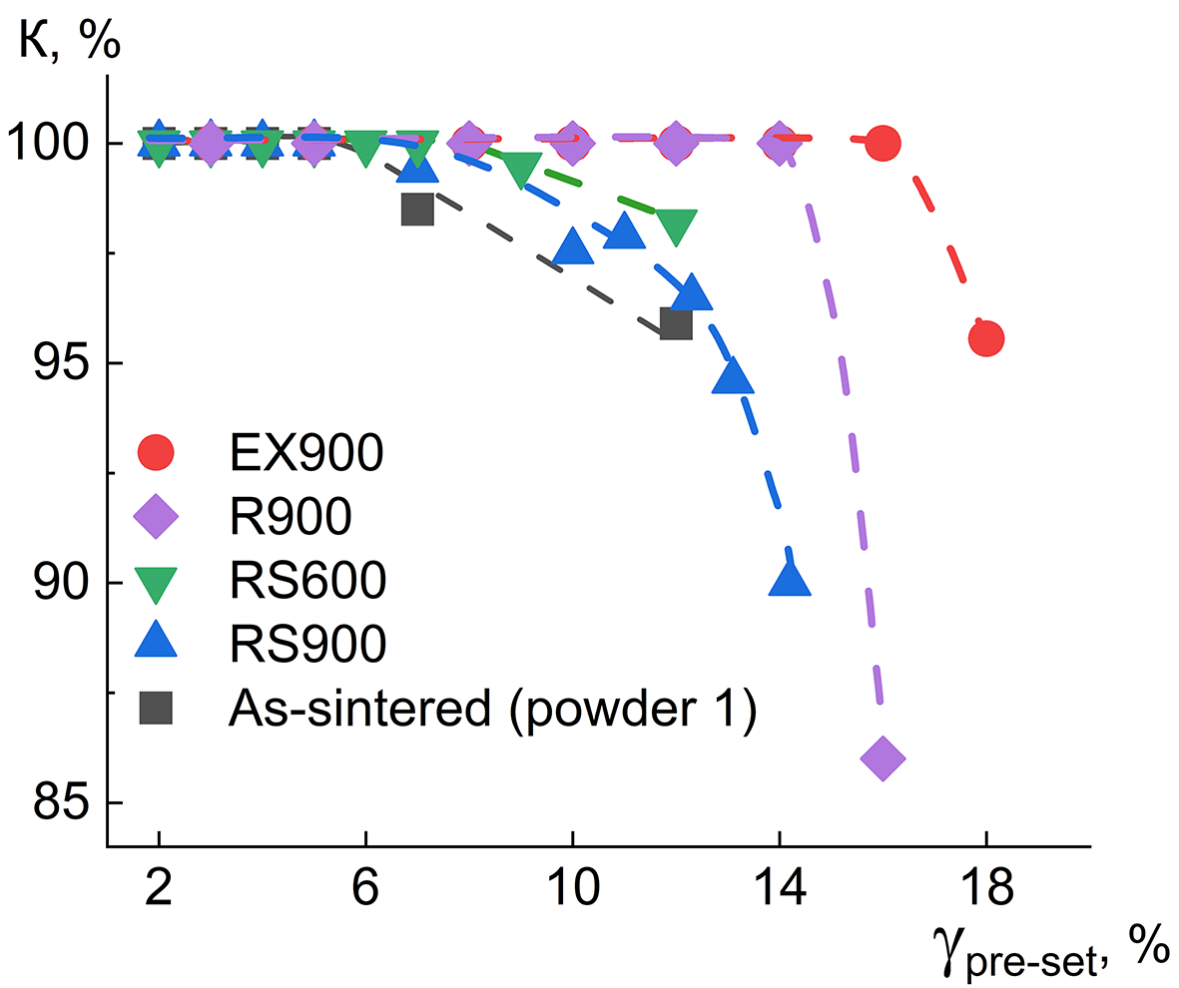
Shape recovery coefficient (K) depending on a pre-set strain ($\:{\gamma\:}_{pre-set})$.

Figure 6 shows the dependence of the K value on the pre-set strain of the samples. The K value was calculated using both superelastic strain and strain recovered at heating like it is schematically shown in Fig. S1b. The samples R900 and EX900 have the highest critical deformation of 14 and 16%, which is probably due to the same reason as for their highest recoverable deformation under superelasticity: the highest yield point among other samples, due to which large deformations are recovered via superelasticity mechanism.

**Fig. 7:**
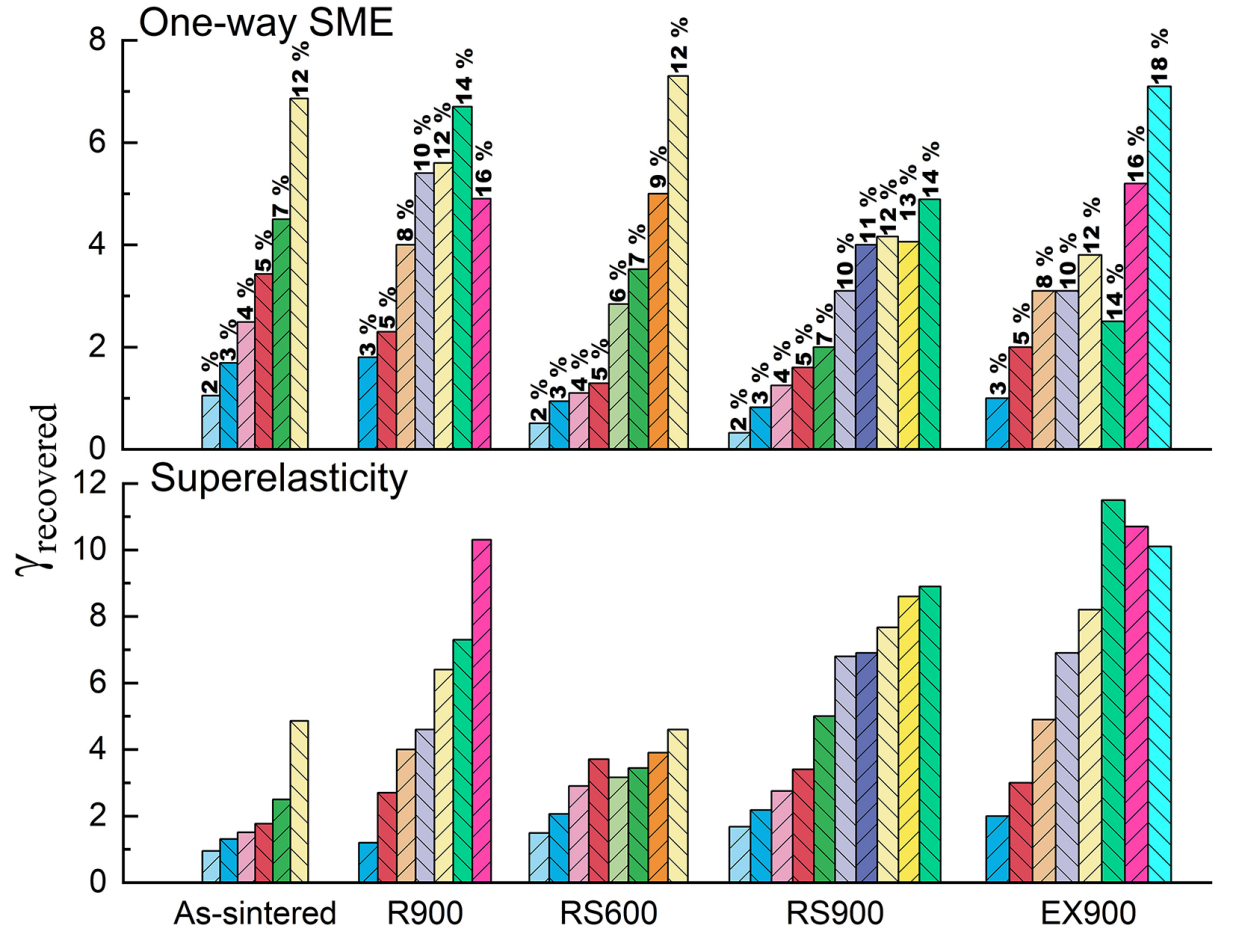
Parts of deformation recovered by superelasticity and one-way shape memory effect for a certain pre-set strain induced by torsion. The bars show how much strain is recovered by a certain mechanism (SME and SE) for each pre-set strain. Numbers above the bars show a certain pre-set strain.

Figure 7 shows the strain recovered by superelasticity and one-way shape memory effect mechanisms for each sample at all pre-set strains.

The maximum strain recovered during one-way SME was about 7%, except for swaging at 900 °C. However, only samples after swaging and radial shear rolling demonstrate a narrow range of shape recovery. Typically, cast NiTi alloys subjected to various treatments demonstrate recovery strain from 7 to 16% via different recovery mechanisms and depending on load type under testing (see Table S3 in the supplementary file)^1,27–30^. In turn, NiTi alloys in the current study demonstrate superelastic strain up to 16% and one-way shape memory effect up to 7%. However, torsional deformation we used for testing has not been applied previously to the best of our knowledge, and this fact makes the comparison difficult but brings new data.

The results obtained make it possible to select the optimal thermomechanical processing technology used in the work to ensure the maximum level of functional properties, depending on the main criterion of functionality. If superelasticity is required from a structural element, then, depending on the level of deformation during operation, it is necessary to use radial shear rolling or extrusion technologies, which in turn provide the highest possible critical deformation in the B2 state. If a one-way shape memory effect is required, then the optimal technology is swaging and radial shear rolling, since, in combination with high functional properties, samples obtained using these methods have a relatively narrow shape recovery interval.

## Conclusion

Our findings have shown that the martensitic transformation and the characteristics of superelasticity and one-way shape memory effect depend on deformation methods. It has been established that the use of radial shear rolling and extrusion makes it possible to achieve a high level of superelasticity (14%) under inducing torsional strain in B2 state. Under specified deformation in the B2-phase + martensite state, the alloys demonstrate superelasticity (up to ~ 10%) and a one-way shape memory effect (up to ~ 7%). In turn, only radial shear rolling and rotary swaging provide a high level of reversible strain upon a one-way shape memory effect (~ 5–7%) in combination with a narrow shape recovery interval (40–50 °C). The maximum critical strain recovered by a combination of superelasticity and one-way shape memory effect has been found to be 14 and 16% for samples subjected to rolling and extrusion.

It is shown that the width of the temperature interval of martensitic transformation correlates with the integral broadening of the Bragg reflection peak (101) of the B2-phase. Only rotary swaging and radial shear rolling lead to a decrease in integral broadening and give narrow temperature intervals of martensitic transformation and accordingly narrow temperature intervals of strain recovery. Extrusion virtually does not lead to a decrease in integral broadening, and the as-sintered samples with high heterogeneity, as well as ones after extrusion, demonstrate a multi-stage martensitic transformation with wide temperature intervals of its realization. Consequently, only radial shear rolling and rotary swaging cause the redistribution of elements in the matrix and lead to homogenization.

Thus, conventional hot deformation methods in combination with powder technology can yield a product with relatively high functional characteristics. It may be a promising technology for the semi-industrial manufacturing of NiTi rods for various applications.

## Electronic supplementary material

Below is the link to the electronic supplementary material.

Supplementary Material 1

## Data Availability

The datasets used and/or analysed during the current study available from the corresponding author on reasonable request.
