# Supplementary material for: Torsional behavior of Ni-rich NiTi alloys obtained by powder metallurgy and hot deformation
Source: Sci Rep. 2024 Nov 18;14:28431. doi: 10.1038/s41598-024-79545-2 (PMC11574033; doi:10.1038/s41598-024-79545-2)
Supplement: Supplementary file 1 — Supplementary Material 1 [file 41598_2024_79545_MOESM1_ESM.docx]

**Torsional behavior of Ni-rich NiTi alloys obtained by powder metallurgy and hot deformation**

Sergey Volodko^1^, Galina Markova^2^, Sergey Yudin^1^, Darya Permyakova^2^, ­Ivan Alimov^2,3^_,_ Evgeny Evstratov^4^, Dmitry Moskovskikh^5^, Alexander Khort^6^, Anatoly Kasimtsev^3^

*^1^ Moscow Polytechnic University, Moscow, Russia*

*^2^ Tula state university, Tula, Russia*

*^3^ LLC Metsintez, Tula, Russia*

*^4^ A. Baikov Institute of Metallurgy and Materials Science, Moscow, Russia*

*^5^ National University of Science and Technology MISIS, Moscow, Russia*

*^6^ KTH Royal Institute of Technology, Stockholm, Sweden*

**Corresponding authors.*

*E-mail addresses: volodko.sv@yandex.ru (SV),* [*mos@misis.ru*](mailto:mos@misis.ru) *(DM), khort@kth.se (KA)*

**Deformation procedure.** *For radial shear rolling, sintered blanks of a diameter of 33 mm and length 200 mm were used. The rolling process is a special case of skew rolling when a mandrel is used to produce tubes, but specially developed for the deformation of solid rods [Galkin S.P., Romantsev B.A., Kharitonov E.A. Putting into practice innovative potential in the universal radial-shear rolling process // CIS Iron and Steel Review. 2014. Vol. 2014. P. 35 – 39]. Radial shear rolling was carried out in two stages. In the first stage, four passes were made with a true deformation in each pass of e = 0.1; 0.3; 0.6 and 0.8 (rod diameters were 30, 24, 18, and 15 mm, respectively). The heating temperature of the workpiece for deformation in first stage was 1000 °C. In the second stage, 5 passes were made with a true deformation of e = 0.9; 1.0; 1.1; 1.2; 1.4 (rod diameters were 13, 12, 11, 10, and 8 mm, respectively). The heating temperature of the workpiece for deformation in second stage was 900 °C. Between passes the workpiece was placed in a furnace to heat it up to deformation temperature. The strain rate for rolling can be approximately assessed as around 3 s^-1^.*

*Extrusion was performed on a horizontal hydraulic press P8041 with a maximum force of 1250 tons. A container with a diameter of 80 mm and a matrix with a conical entry funnel (cone angle of 120°) and an opening diameter of 35 mm were used. The sintered blank had a diameter of 78 mm and a length of 174 mm. Extrusion was performed from a diameter of 78 mm to 35 mm in one pass with a true deformation of e = 0.8 (the extrusion ratio was 5.2).* *The heating temperature for deformation was 900 °C. The heating was performed in a hydrogen atmosphere. The strain rate for extrusion is around 30 s^-1^.*

*Rotary swaging was conducted for the sintered blanks of a diameter of 15 mm, length 200–220 mm. Rotary swaging was carried out at temperatures of 600 and 900 with a true deformation of e=0.6 (rod diameter after deformation – 8 mm) on a modernized rotary forging machine RKM2 B2129.02 with two forging dies. The workpiece was heated up 5 times up to deformation temperature between passes during swaging*. *The strain rate for swaging is around 150 s^-1^.*


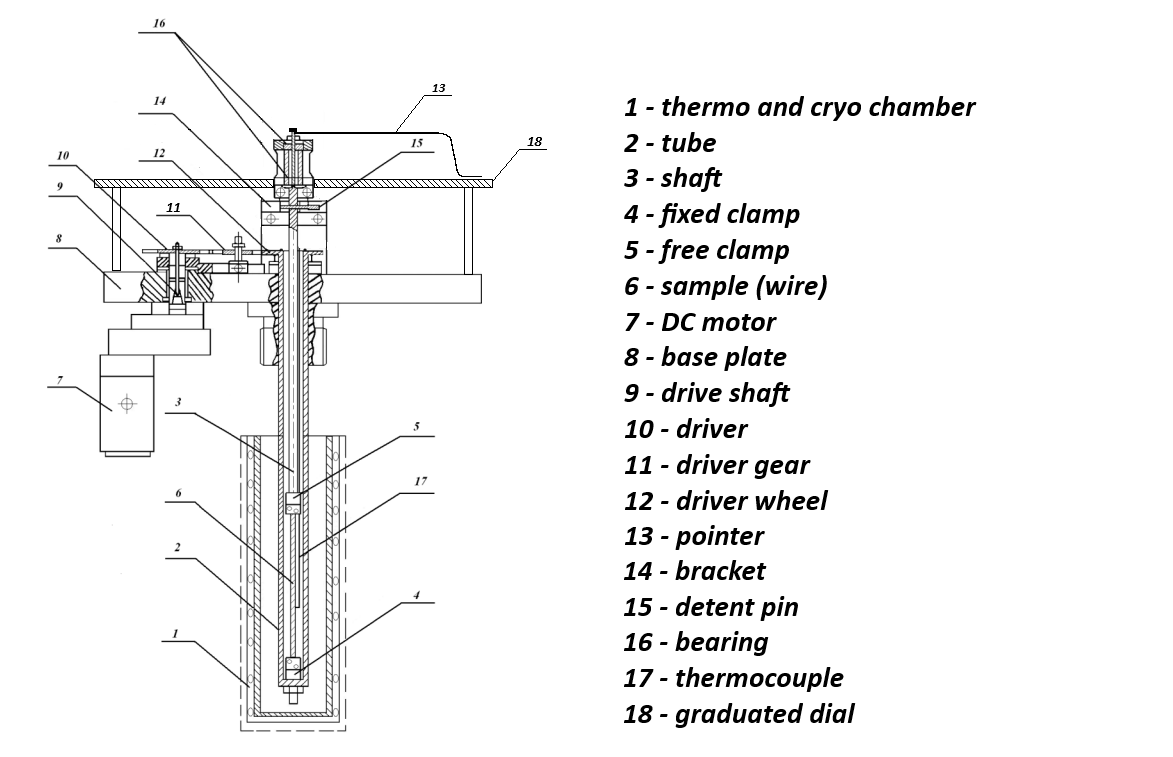


a)


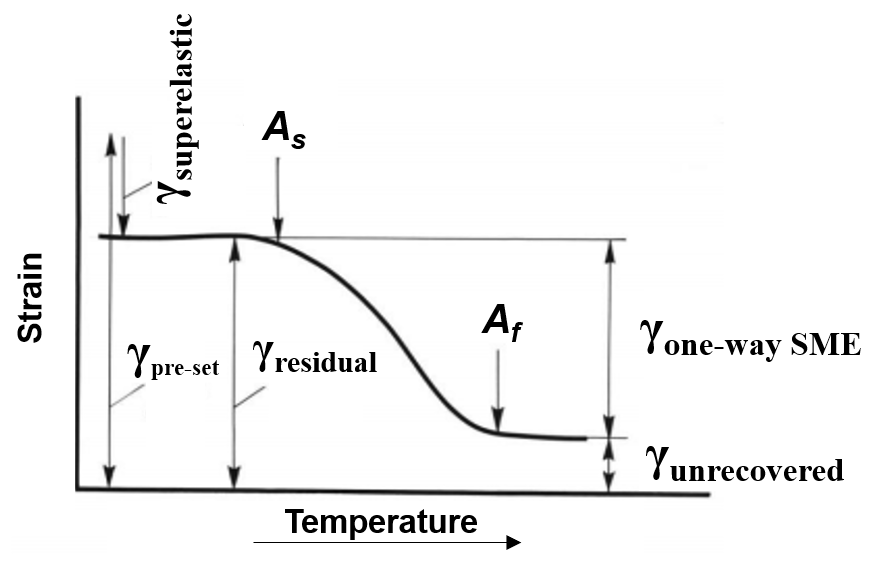


b)

Fig. S1. The installation for the evaluation of shape memory characteristics via torsional load (a) and schematic representation of the assessment of shape memory effect and superelasticity (b) and.

Table S1. Chemical composition of the NiTi powder alloys

| Base elements, wt% | | Impurities, wt% | | | | | | | |
| --- | --- | --- | --- | --- | --- | --- | --- | --- | --- |
| Ni | Ti | C | Co | Fe | Si | N | O | H | Ca |
| Powder 1^**^ | | | | | | | | | |
| 55.5 | Res. | 0.067 | - | 0.1 | 0.02 | 0.015 | 0.1 | 0.0028^*^ | 0.13 |
| Powder 2^***^ | | | | | | | | | |
| 55.4 | Res. | 0.061 | - | 0.075 | 0.02 | 0.025 | 0.074 | 0.0031^*^ | 0.11 |

*After vacuum annealing;

** Powder 1 was used for rotary swaging and radial shear rolling;

*** Powder 2 was used for extrusion.

**** Powder 1 and powder 2 are the same charge composition, but two different batches of the powder obtained via the calciothermic synthesis. The small difference in the content of main elements demonstrates the reproducibility of the obtaining method.

Table S2. Mechanical properties of the NiTi powder alloys

|  | Deformation temperature and strain | UTS,  MPa | YS,  MPa | Elongation,  % | E,  GPa | Hardness,  HV | Grain size, µm |
| --- | --- | --- | --- | --- | --- | --- | --- |
| As-sintered state  Powder 1  Powder 2 | - | 980  1078 | 480  422 | 3.6  4.1 | 45  57 | 420±15  399±4 | 92±7  150±5 |
| Rotary swaging  (Powder 1) | 900 °С, *е* = 0.6  600 °С, *е* = 0.6 | 1390  820 | 600  480 | 14.5  17.8 | 53  69 | 398±5  295±4 | 38±4  4±1 |
| Radial shear rolling (Powder 1) | 1000 °С, *е* = 0.1  1000 °С, *е* = 0.3  1000 °С, *е* = 0.6  1000 °С, *е* = 0.8  900 °С, *е* = 1.4 | -  -  -  1140  1160 | -  -  -  820  808 | -  -  -  5.1  11.3 | -  -  -  77  89 | 365±6  386±10  400±8  384±8  419±7 | 92±3  98±3  72±4  64±2  34±2 |
| Extrusion  (Powder 2) | 900 °С, *е* = 0.8 | 1250 | 780 | 13.1 | 68 | 419±10 | 32±2 |

Notation: ***e*** stands as strain and was estimated as the natural logarithm of the ratio between diameters before and after deformation. The mechanical properties were measured at room temperature using an Instron 5581 tensile testing machine at a strain rate of 10^-3^ s^-1^ on cylindrical samples with a length of reduced section of 25 mm and diameter of 5 mm. Hardness values were obtained at a load of 100 g (0.98 N) by Vickers hardness test according to 50 measurements for each sample. Grain sizes were assessed via metallographic analysis using ImageJ software (250 grains for each sample).


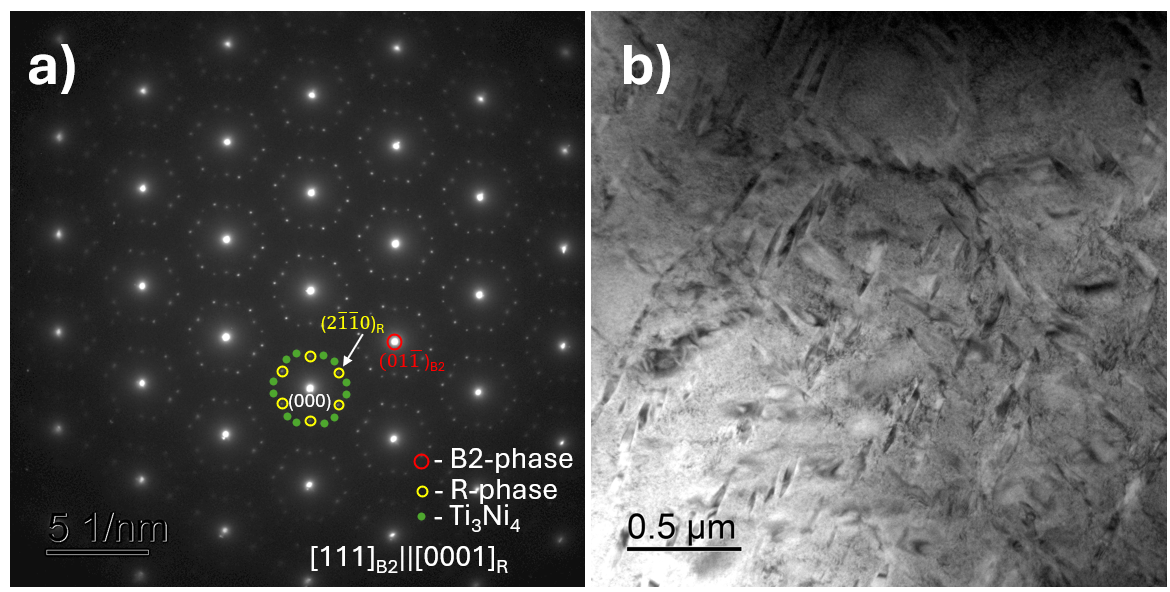


Figure S2. SAED (a) and corresponding BF image (b) taken at room temperature of the NiTi alloy subjected to radial shear rolling. Figure (b) represents the matrix with Ti_3_Ni_4_ particles of needle type and R-martensite. The presence of these phases confirmed by SAED in Figure (a). Correspondingly, the nucleation of Ti_3_Ni_4_ precipitates provides a multi-stage transformation in alloys after deformation cooled to room temperature in air with no additional heat treatment. Transmission electron microscopy was performed on a JEM-2100 microscope at an acceleration voltage of 200 kV on samples subjected to radial shear rolling with no additional heat treatment.

| 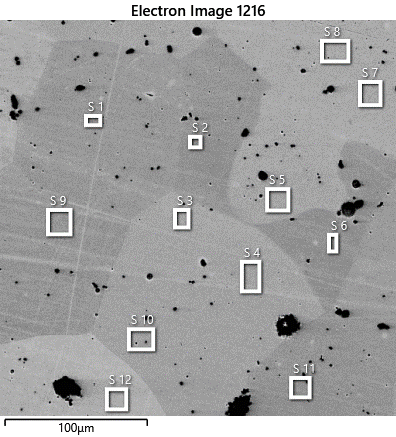 | Total table %at   \| Spectrum Label \| Ti \| Ni \| Total \| \| --- \| --- \| --- \| --- \| \| S 1 \| 49.6 \| 50.4 \| 100 \| \| S 2 \| 49.89 \| 50.11 \| 100 \| \| S 3 \| 49.8 \| 50.2 \| 100 \| \| S 4 \| 49.72 \| 50.28 \| 100 \| \| S 5 \| 50.06 \| 49.94 \| 100 \| \| S 6 \| 49.72 \| 50.28 \| 100 \| \| S 7 \| 49.5 \| 50.5 \| 100 \| \| S 8 \| 49.68 \| 50.32 \| 100 \| \| S 9 \| 49.47 \| 50.53 \| 100 \| \| S 10 \| 49.51 \| 50.49 \| 100 \| \| S 11 \| 50.43 \| 49.57 \| 100 \| \| S 12 \| 49.58 \| 50.42 \| 100 \| |
| --- | --- | --- | --- | --- | --- | --- | --- | --- | --- | --- | --- | --- | --- | --- | --- | --- | --- | --- | --- | --- | --- | --- | --- | --- | --- | --- | --- | --- | --- | --- | --- | --- | --- | --- | --- | --- | --- | --- | --- | --- | --- | --- | --- | --- | --- | --- | --- | --- | --- | --- | --- | --- | --- |
| 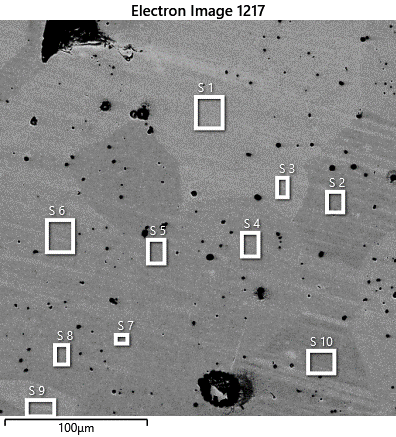 | Total table %at   \| Spectrum Label \| Ti \| Ni \| Total \| \| --- \| --- \| --- \| --- \| \| S 1 \| 49.77 \| 50.23 \| 100 \| \| S 2 \| 50.71 \| 49.29 \| 100 \| \| S 3 \| 49.73 \| 50.27 \| 100 \| \| S 4 \| 49.99 \| 50.01 \| 100 \| \| S 5 \| 49.89 \| 50.11 \| 100 \| \| S 6 \| 49.83 \| 50.17 \| 100 \| \| S 7 \| 49.77 \| 50.23 \| 100 \| \| S 8 \| 49.62 \| 50.38 \| 100 \| \| S 9 \| 49.76 \| 50.24 \| 100 \| \| S 10 \| 49.44 \| 50.56 \| 100 \| |
| 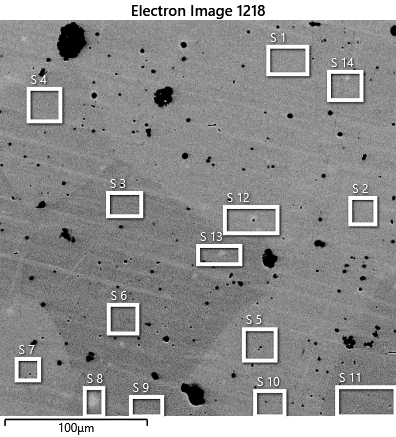 | Total table %at   \| Spectrum Label \| Ti \| Ni \| Total \| \| --- \| --- \| --- \| --- \| \| S 1 \| 50.24 \| 49.76 \| 100 \| \| S 2 \| 49.53 \| 50.47 \| 100 \| \| S 3 \| 49.36 \| 50.64 \| 100 \| \| S 4 \| 49.81 \| 50.19 \| 100 \| \| S 5 \| 49.24 \| 50.76 \| 100 \| \| S 6 \| 49.06 \| 50.94 \| 100 \| \| S 7 \| 49.63 \| 50.37 \| 100 \| \| S 8 \| 49.61 \| 50.39 \| 100 \| \| S 9 \| 50.03 \| 49.97 \| 100 \| \| S 10 \| 49.77 \| 50.23 \| 100 \| \| S 11 \| 49.79 \| 50.21 \| 100 \| \| S 12 \| 49.61 \| 50.39 \| 100 \| \| S 13 \| 49.82 \| 50.18 \| 100 \| \| S 14 \| 49.33 \| 50.67 \| 100 \| |
|  |  |

Figure S3. EDS results of the as-sintered NiTi alloy fabricated from Powder 1 (see Table S1). The difference ($C_{max}-C_{min})$ in Ni concentration is 1.6 %at. Black areas in the electron images are TiO_x_ inclusions and pores (see Figure S9). TiO_x_ inclusions may be due to the interaction of oxygen with metal matrix in closed pores during vacuum sintering. To avoid it and decrease oxygen content, the vacuum annealing of a blank after isostatic pressing may be useful while the porosity is still open.

| 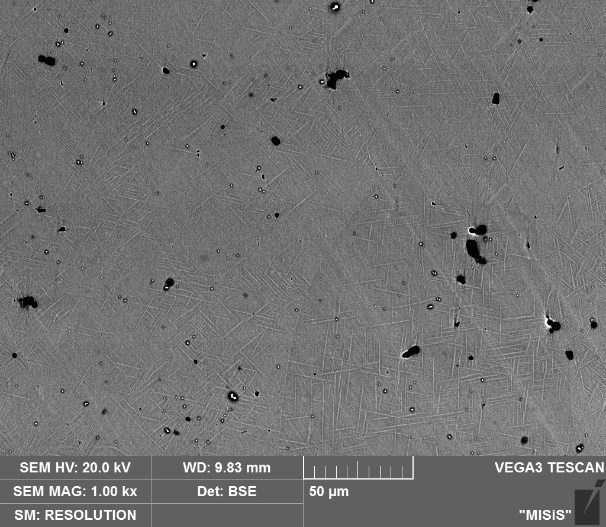 | 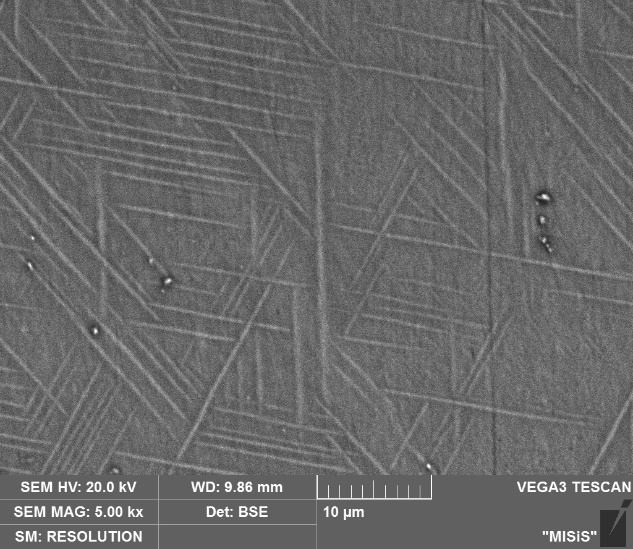 |
| --- | --- |

Figure S4. Microstructure of the as-sintered NiTi alloy fabricated from Powder 2 (see Table S1). EDS analysis was not performed.

| 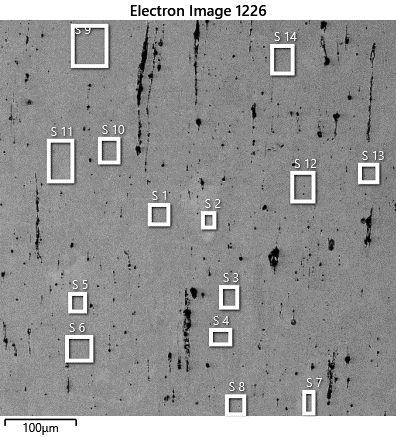 | Total table %at   \| Spectrum Label \| Ti \| Ni \| Total \| \| --- \| --- \| --- \| --- \| \| S 1 \| 49.4 \| 50.6 \| 100 \| \| S 2 \| 49.41 \| 50.59 \| 100 \| \| S 3 \| 49.52 \| 50.48 \| 100 \| \| S 4 \| 49.33 \| 50.67 \| 100 \| \| S 5 \| 49.67 \| 50.33 \| 100 \| \| S 6 \| 49.42 \| 50.58 \| 100 \| \| S 7 \| 49.39 \| 50.61 \| 100 \| \| S 8 \| 49.41 \| 50.59 \| 100 \| \| S 9 \| 49.61 \| 50.39 \| 100 \| \| S 10 \| 49.57 \| 50.43 \| 100 \| \| S 11 \| 49.28 \| 50.72 \| 100 \| \| S 12 \| 49.23 \| 50.77 \| 100 \| \| S 13 \| 49.38 \| 50.62 \| 100 \| \| S 14 \| 49.41 \| 50.59 \| 100 \| |
| --- | --- | --- | --- | --- | --- | --- | --- | --- | --- | --- | --- | --- | --- | --- | --- | --- | --- | --- | --- | --- | --- | --- | --- | --- | --- | --- | --- | --- | --- | --- | --- | --- | --- | --- | --- | --- | --- | --- | --- | --- | --- | --- | --- | --- | --- | --- | --- | --- | --- | --- | --- | --- | --- | --- | --- | --- | --- | --- | --- | --- | --- |
|  |  |
| 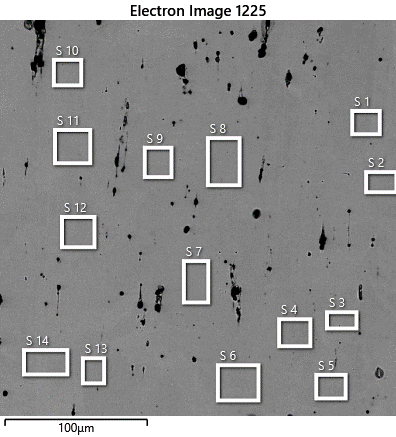 | Total table %at   \| Spectrum Label \| Ti \| Ni \| Total \| \| --- \| --- \| --- \| --- \| \| S 1 \| 49.38 \| 50.62 \| 100 \| \| S 2 \| 49.11 \| 50.89 \| 100 \| \| S 3 \| 49.42 \| 50.58 \| 100 \| \| S 4 \| 49.59 \| 50.41 \| 100 \| \| S 5 \| 49.29 \| 50.71 \| 100 \| \| S 6 \| 49.4 \| 50.6 \| 100 \| \| S 7 \| 49.44 \| 50.56 \| 100 \| \| S 8 \| 49.33 \| 50.67 \| 100 \| \| S 9 \| 49.26 \| 50.74 \| 100 \| \| S 10 \| 49.43 \| 50.57 \| 100 \| \| S 11 \| 49.36 \| 50.64 \| 100 \| \| S 12 \| 49.58 \| 50.42 \| 100 \| \| S 13 \| 49.17 \| 50.83 \| 100 \| \| S 14 \| 49.12 \| 50.88 \| 100 \| |

Figure S5. EDS results of the NiTi alloy after radial shear rolling. The difference ($C_{max}-C_{min})$ in Ni concentration is 0.6 %at. Black areas in the electron images are pores and sometimes the inclusion bands of CaO and TiO_x_ (see Figure S10). The former is a by-product after synthesis and presence in the structure in minor quantities, and the latter may be a consequence of metallographic sample preparation or is inherited from the as-sintered structure.

| 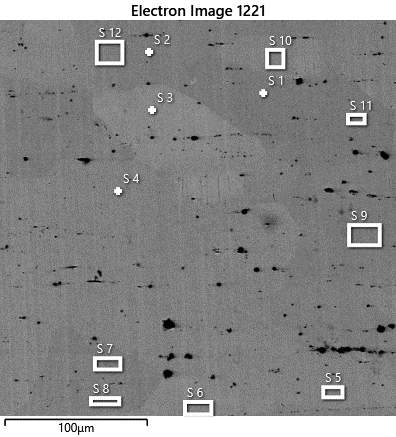 | Total table %at   \| Spectrum Label \| Ti \| Ni \| Total \| \| --- \| --- \| --- \| --- \| \| S 1 \| 49.34 \| 50.66 \| 100 \| \| S 2 \| 49.1 \| 50.9 \| 100 \| \| S 3 \| 49.15 \| 50.85 \| 100 \| \| S 4 \| 49.6 \| 50.4 \| 100 \| \| S 5 \| 49.3 \| 50.7 \| 100 \| \| S 6 \| 49.65 \| 50.35 \| 100 \| \| S 7 \| 49.53 \| 50.47 \| 100 \| \| S 8 \| 49.26 \| 50.74 \| 100 \| \| S 9 \| 49.71 \| 50.29 \| 100 \| \| S 10 \| 48.98 \| 51.02 \| 100 \| \| S 11 \| 49.14 \| 50.86 \| 100 \| \| S 12 \| 49.19 \| 50.81 \| 100 \| |
| --- | --- | --- | --- | --- | --- | --- | --- | --- | --- | --- | --- | --- | --- | --- | --- | --- | --- | --- | --- | --- | --- | --- | --- | --- | --- | --- | --- | --- | --- | --- | --- | --- | --- | --- | --- | --- | --- | --- | --- | --- | --- | --- | --- | --- | --- | --- | --- | --- | --- | --- | --- | --- | --- |
| 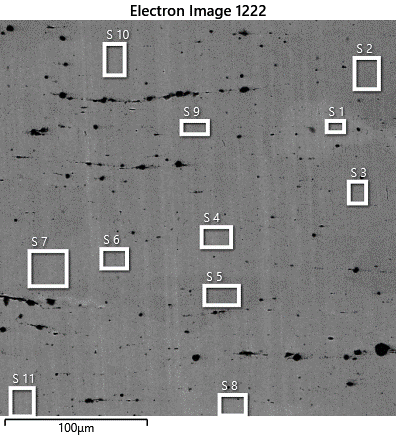 | Total table %at   \| Spectrum Label \| Ti \| Ni \| Total \| \| --- \| --- \| --- \| --- \| \| S 1 \| 49.38 \| 50.62 \| 100 \| \| S 2 \| 49.34 \| 50.66 \| 100 \| \| S 3 \| 49.37 \| 50.63 \| 100 \| \| S 4 \| 49.37 \| 50.63 \| 100 \| \| S 5 \| 49.33 \| 50.67 \| 100 \| \| S 6 \| 49.04 \| 50.96 \| 100 \| \| S 7 \| 49.25 \| 50.75 \| 100 \| \| S 8 \| 49.16 \| 50.84 \| 100 \| \| S 9 \| 49.26 \| 50.74 \| 100 \| \| S 10 \| 49.15 \| 50.85 \| 100 \| \| S 11 \| 48.96 \| 51.04 \| 100 \| |
| 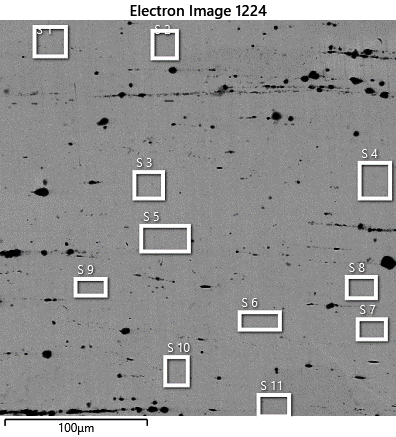 | Total table %at   \| Spectrum Label \| Ti \| Ni \| Total \| \| --- \| --- \| --- \| --- \| \| S 1 \| 49 \| 51 \| 100 \| \| S 2 \| 48.96 \| 51.04 \| 100 \| \| S 3 \| 48.89 \| 51.11 \| 100 \| \| S 4 \| 49 \| 51 \| 100 \| \| S 5 \| 49.03 \| 50.97 \| 100 \| \| S 6 \| 49.23 \| 50.77 \| 100 \| \| S 7 \| 49.24 \| 50.76 \| 100 \| \| S 8 \| 49.12 \| 50.88 \| 100 \| \| S 9 \| 49.11 \| 50.89 \| 100 \| \| S 10 \| 49.5 \| 50.5 \| 100 \| \| S 11 \| 49.38 \| 50.62 \| 100 \| |
|  |  |

Figure S6. EDS results of the NiTi alloy after rotary swagging at 900 °C. The difference ($C_{max}-C_{min})$ in Ni concentration is 0.8 %at. Black areas in the electron images are pores and sometimes the inclusion bands of CaO and TiO_x_. The former is a by-product after synthesis and presence in the structure in minor quantities, and the latter may be a consequence of metallographic sample preparation.

| 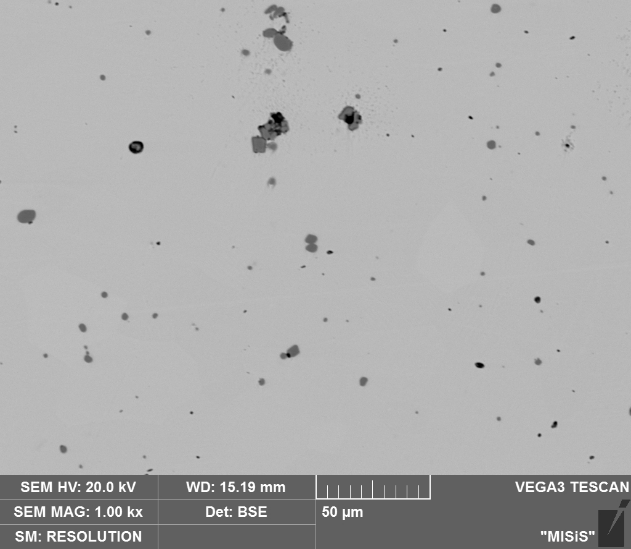 | EDS analysis was not performed |
| --- | --- |
| 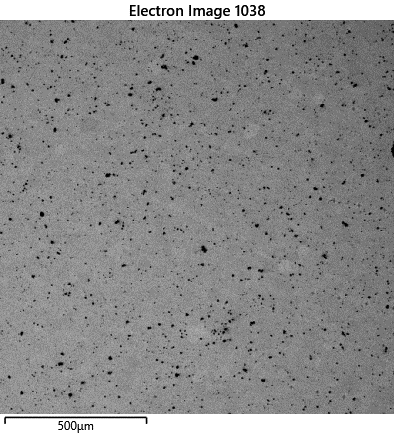 | Results of an integral EDS analysis  Total table % at   \| Spectrum Label \| Ti \| Ni \| Total \| \| --- \| --- \| --- \| --- \| \| D 2 \| 49.63 \| 50.37 \| 100 \| |

Figure S7. Microstructure of the NiTi alloy after extrusion at 900 °C.

| 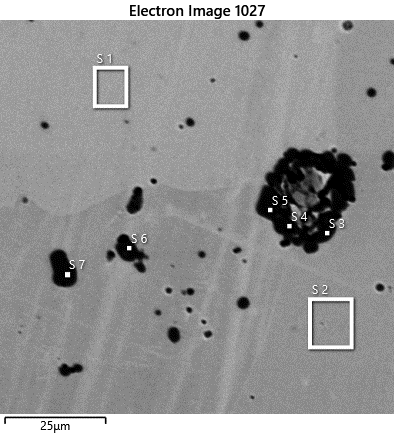 | Total table % at   \| Spectrum Label \| O \| Ca \| Ti \| Ni \| Total \| \| --- \| --- \| --- \| --- \| --- \| --- \| \| S 1 \|  \|  \| 49.5 \| 50.48 \| 100 \| \| S 2 \|  \|  \| 49.7 \| 50.29 \| 100 \| \| S 3 \| 31.34 \| 0.25 \| 66.9 \| 1.48 \| 100 \| \| S 4 \| 37.26 \|  \| 59.6 \| 3.18 \| 100 \| \| S 5 \| 33.46 \| 0.16 \| 65.5 \| 0.89 \| 100 \| \| S 6 \| 30.75 \|  \| 68.1 \| 1.12 \| 100 \| |
| --- | --- | --- | --- | --- | --- | --- | --- | --- | --- | --- | --- | --- | --- | --- | --- | --- | --- | --- | --- | --- | --- | --- | --- | --- | --- | --- | --- | --- | --- | --- | --- | --- | --- | --- | --- | --- | --- | --- | --- | --- | --- | --- | --- |

Figure S8. EDS results of inclusions in the as-sintered NiTi (Powder 1).

| 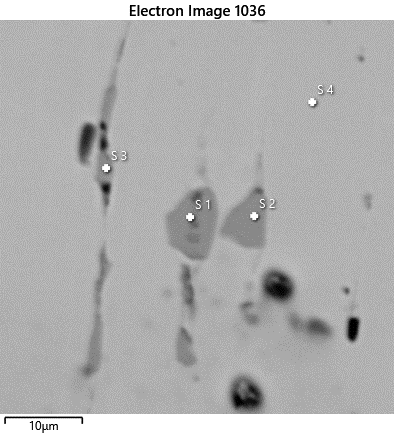  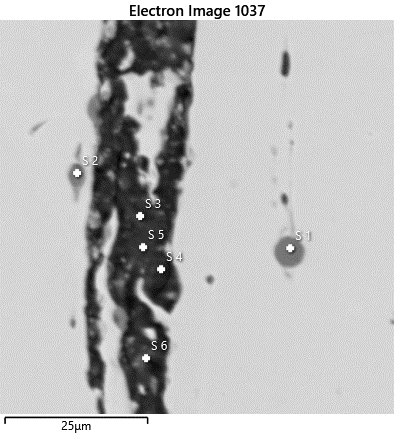 | Total table % at   \| Spectrum Label \| Ti \| Ni \| Total \| \| --- \| --- \| --- \| --- \| \| S 1 \| 62.9 \| 37.1 \| 100 \| \| S 2 \| 66.91 \| 33.09 \| 100 \| \| S 3 \| 65.66 \| 34.34 \| 100 \| \| S 4 \| 49.52 \| 50.48 \| 100 \|   Total table % at   \| Spectrum Label \| O \| Ca \| Ti \| Fe \| Ni \| Total \| \| --- \| --- \| --- \| --- \| --- \| --- \| --- \| \| S 1 \| 30.03 \| 0.16 \| 69 \|  \| 0.85 \| 100 \| \| S 2 \| 30.79 \| 0.41 \| 67.7 \|  \| 1.09 \| 100 \| \| S 3 \| 74.02 \| 24.71 \| 0.42 \| 0.45 \| 0.41 \| 100 \| \| S 4 \| 75.63 \| 20.68 \| 1.2 \| 1.94 \| 0.55 \| 100 \| \| S 5 \| 73.56 \| 25.36 \| 0.3 \| 0.37 \| 0.41 \| 100 \| \| S 6 \| 71.54 \| 18.11 \| 4.56 \| 1.23 \| 4.57 \| 100 \| |
| --- | --- | --- | --- | --- | --- | --- | --- | --- | --- | --- | --- | --- | --- | --- | --- | --- | --- | --- | --- | --- | --- | --- | --- | --- | --- | --- | --- | --- | --- | --- | --- | --- | --- | --- | --- | --- | --- | --- | --- | --- | --- | --- | --- | --- | --- | --- | --- | --- | --- | --- | --- | --- | --- | --- | --- | --- | --- | --- | --- | --- | --- | --- | --- | --- | --- | --- | --- | --- | --- | --- |
| Figure S9. EDS results of inclusions in the NiTi alloy after radial shear rolling at 900 °C. | |
| 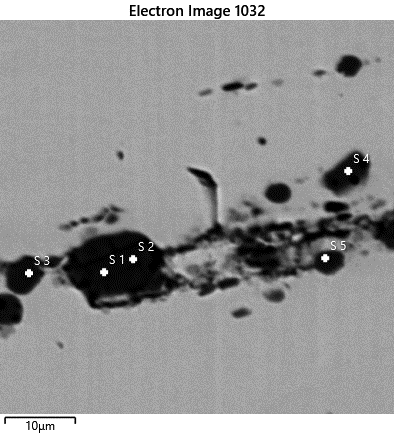  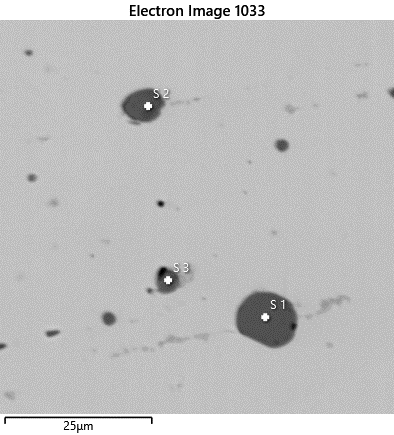 | Total table % at   \| Spectrum Label \| O \| Ca \| Ti \| Ni \| Total \| \| --- \| --- \| --- \| --- \| --- \| --- \| \| S 1 \| 31.09 \| 0.42 \| 67.4 \| 1.07 \| 100 \| \| S 2 \| 35.17 \| 0.44 \| 63.6 \| 0.8 \| 100 \| \| S 3 \| 29.89 \|  \| 68.9 \| 1.17 \| 100 \| \| S 4 \| 31.21 \| 0.73 \| 67.1 \| 1 \| 100 \| \| S 5 \| 28.36 \| 0.77 \| 66.1 \| 4.76 \| 100 \|   Total table % at   \| Spectrum Label \| N \| O \| Ca \| Ti \| Ni \| Total \| \| --- \| --- \| --- \| --- \| --- \| --- \| --- \| \| S 1 \| 14.2 \| 25.66 \|  \| 59.4 \| 0.74 \| 100 \| \| S 2 \|  \| 31.69 \| 0.37 \| 66.97 \| 0.96 \| 100 \| \| S 3 \|  \| 31.34 \| 0.33 \| 66.21 \| 2.12 \| 100 \| |
| Figure S10. EDS results of inclusions in the NiTi alloy after rotary swaging at 900 °C. | |
| 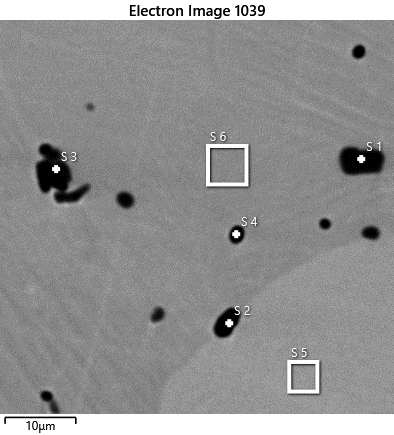 | Total table % at   \| Spectrum Label \| O \| Ca \| Ti \| Cr \| Ni \| Total \| \| --- \| --- \| --- \| --- \| --- \| --- \| --- \| \| S 1 \| 28.81 \|  \| 68.7 \|  \| 2.49 \| 100 \| \| S 2 \|  \|  \| 45 \| 9.32 \| 45.65 \| 100 \| \| S 3 \| 41.83 \| 0.49 \| 35.2 \| 9.89 \| 12.62 \| 100 \| \| S 4 \|  \|  \| 48.9 \| 0.52 \| 50.64 \| 100 \| \| S 5 \|  \|  \| 49.5 \|  \| 50.5 \| 100 \| \| S 6 \|  \|  \| 49.3 \|  \| 50.68 \| 100 \| |
| Figure S11. EDS results of inclusions in the NiTi alloy after extrusion at 900 °C. Chromium was observed because the metallographic sample was prepared with using a Cr_2_O_3_ powder for polishing. | |

Table S3. Functional properties of NiTi alloys prepared by different processes

| No. | Composition | Recovery strain****, % | Deformation scheme | Manufacturing process | State | Ref. |
| --- | --- | --- | --- | --- | --- | --- |
| 1 | Ti-50.5 %at Ni (Powder 1) | 7*** (11.5 max out of 12)  12**  7*** | Torsion | Powder metallurgy | As-sintered | The current study |
|  |  | 14*  14**  7*** |  |  | Radial shear rolling at 900 °C + cooling in air |  |
|  |  | 7***  12**  7*** |  |  | Rotary swaging at 900 °C + cooling in air |  |
|  | Ti-50.4 %at Ni (Powder 2) | 16*  14**  7*** |  |  | Extrusion at 900 °C + cooling in air |  |
| 2 | Ti-50.7 %at Ni | 16* | Bending | Casting | Cold drawing + recrystallization annealing 600- 700 °C (water quenched) + aging at 430 °C for 1-10 h | [1] |
| 3 | Ti-50.8 %at Ni | 7** | Tension | Casting | Cold rolling (10 %) + annealing at 600 °C for 30 min (water quenched) | [2] |
| 4 | Ti-50.0 %at Ni | 6.8*** | Bending | Casting | Rotary forging at 800-900 °C, annealing at 700 for 30 min (water quenched) + torsional deformation at 500 °C under 0.1 s^-1^ (14 and 30 turns) up to e = 4.3 and 9.1 | [3] |
| 5 | Ti-50.2 %at Ni | 5***  (9.3* max out of 10) | Bending | Laser powder bed fusion | Thermocycling and annealing at 350-400 °C for an hour | [4] |
| 6 | Ti-50.9 %at Ni | 6.5** | Tension | Casting | Annealing at 800 °C for 1 h and cold rolled to 42 % then annealed at 250-600 °C for 2-10 min | [5] |

* - induced deformation was recovered via the combination of several following mechanisms: superelasticity, one-way shape memory effect, elastic deformation, elastic twinning.

** - induced deformation was recovered via elastic / elastic twinning + superelesticity.

*** - induced deformation was recovered via elastic / elastic twinning + one-way shape memory effect.

**** - recovered strains represented in the table were taken as fully recovered strain with no residual strain that implies 100 % recovery ratio. Exclusions are provided in brackets.

1. Polyakova, K.A., Ryklina, E.P. & Prokoshkin, S.D. Effect of Grain Size and Ageing-Induced Microstructure on Functional Characteristics of a Ti-50.7 at.% Ni Alloy. *Shap. Mem. Superelasticity* **6**, 139–147 (2020). https://doi.org/chedfe
2. Chen, W.; Xi, R.; Jiang, H.; Li, X.; Dong, G.; Wang, X. Superelasticity of Geometrically Graded NiTi Shape Memory Alloys. *Metals* **2023**, *13*, 1518. <https://doi.org/10.3390/met13091518>
3. Komarov, V.; Karelin, R.; Cherkasov, V.; Yusupov, V.; Korpala, G.; Kawalla, R.; Prahl, U.; Prokoshkin, S. Effect of Severe Torsion Deformation on Structure and Properties of Titanium–Nickel Shape Memory Alloy. *Metals* **2023**, *13*, 1099. <https://doi.org/10.3390/met13061099>
4. Tsaturyants, M., Sheremetyev, V., Dubinskiy, S. *et al.* Structure and Properties of Ti–50.2Ni Alloy Processed by Laser Powder Bed Fusion and Subjected to a Combination of Thermal Cycling and Heat Treatments. *Shap. Mem. Superelasticity* **8**, 16–32 (2022). <https://doi.org/10.1007/s40830-022-00363-4>
5. Aslan Ahadi, Qingping Sun, Stress-induced nanoscale phase transition in superelastic NiTi by in situ X-ray diffraction, Acta Materialia, Volume 90, 2015,Pages 272-281, <https://doi.org/10.1016/j.actamat.2015.02.024>.
